# Comparison of gnotobiotic communities reveals milk-adapted metabolic functions and unexpected amino acid metabolism by the pre-weaning microbiome

**DOI:** 10.1080/19490976.2024.2387875

**Published:** 2024-08-12

**Authors:** Jean-Bernard Lubin, Michael A. Silverman, Paul J. Planet

**Affiliations:** a Division of Infectious Disease, Department of Pediatrics, The Children’s Hospital of Philadelphia, Philadelphia, PA, USA; b Perelman School of Medicine, University of Pennsylvania, Philadelphia, PA, USA; c Department of Microbiology, Perelman School of Medicine, University of Pennsylvania, Philadelphia, PA, USA; d Institute for Immunology and Immune Health, Perelman School of Medicine, University of Pennsylvania, Philadelphia, PA, USA

**Keywords:** Microbiome, gnotobiotics, metabolism

## Abstract

The intestinal microbiome during infancy and childhood has distinct metabolic functions and microbial composition compared to adults. We recently published a gnotobiotic mouse model of the pre-weaning microbiome (PedsCom), which retains a pre-weaning configuration during the transition from a milk-based diet to solid foods, leads to a stunted immune system, and increases susceptibility to enteric infection. Here, we compared the phylogenetic and metabolic relationships of the PedsCom consortium to two adult-derived gnotobiotic communities, Altered Schaedler Flora and Oligo-Mouse Microbiota 12 (Oligo-MM^12^). We find that PedsCom contains several unique functions relative to these adult-derived mouse consortia, including differences in carbohydrate and lipid metabolism genes. Notably, amino acid degradation metabolic modules are more prevalent among PedsCom isolates, which is in line with the ready availability of these nutrients in milk. Indeed, metabolomic analysis revealed significantly lower levels of total free amino acids and lower levels of specific amino acids abundant in milk (e.g. glutamine and glutamic acid) in the intestinal contents of adult PedsCom colonized mice compared to Oligo-MM^12^ controls. Metabolomic analysis of pre-weaning intestinal contents also showed lower levels of amino acids that are replete in milk compared to germ-free controls. Thus, enhanced amino acid metabolism is a prominent feature of the pre-weaning microbiome that may facilitate design of early-life microbiome interventions.

## Introduction

The most dramatic physiological changes in the mammalian microbiome occur during weaning.^1^ The transition from milk to a solid food-based diet is accompanied by radical shifts in intestinal microbiome diversity and composition, as post-weaning associated microbes take advantage of newly available nutrient sources. Disruptions to the early-life microbiome that occur prior to and during weaning have detrimental impacts on host immune system development, increasing the risk of allergy and autoimmunity later in life.^2,3^ To better understand the physiologic impacts of the early-life microbiome, we previously developed a nine-member consortium called Pediatric Community (PedsCom) to model the unique composition and function of mammalian pre-weaning intestinal microbiomes.^4^ This gnotobiotic model is resistant to diet-induced shifts in community structure during weaning. We found that restricting mice to a pre-weaning microbial community in adulthood stunted key markers of immune system maturation (IgA and peripheral regulatory T cells), indicating that development of the microbiome during weaning is crucial for normal immune system maturation. This stunting of the microbiome raises multiple questions about the metabolic capabilities of PedsCom, including which nutrients are used differently relative to adult-derived consortia.

Changes in the types of carbohydrates available during the transition from milk to solid food are often invoked as the key driver of weaning associated changes in the intestinal microbiome.^5^ The carbohydrate fraction of milk is primarily composed of lactose and host-indigestible milk oligosaccharides, while solid foods primarily contain starches and host-indigestible fiber. Although lactose is a major source of available energy in the pre-weaning gut, the majority of this nutrient is consumed and utilized by the host in the small intestine.^6^ As such, in the distal gut, alternate milk-derived carbohydrates such as human milk oligosaccharides (HMOs) likely have more impact on the pre-weaning microbiome. Indeed, HMOs promote neonatal colonization of beneficial species such as *Bifidobacterium* spp.^7,8^

The microbiome changes associated with lipid and protein nutrient composition between pre- and post-weaning diets are less well understood. Lipids are the second most abundant macronutrient after lactose in human milk and provide ~40% of the total energy content.^9^ The protein fraction of milk is another distinguishing factor of pre- and post-weaning nutrient availability. Milk proteins have different amino acid proportions from non-milk sources, containing much higher proline and glutamine content.^10^ Additionally, free amino acids in milk primarily provide glutamic acid, taurine, alanine and glutamine to the pre-weaning gut environment.^11,12^ Overall, proteins are a major source of nitrogen, which is a limiting nutrient in the colonic microbiome,^13^ and there is growing interest in the role of nitrogen sources as drivers of microbiome dynamics.^14^ The differences in the abundance and composition of all three macronutrients (carbohydrate, lipid, and protein) in milk versus solid food likely contribute to the colonization and metabolic output of pre-weaning microbiota.

Here, we compare the phylogenetic diversity and metabolic capabilities of the pre-weaning gnotobiotic community PedsCom to two gnotobiotic communities that model the adult microbiome [Oligo-Mouse Microbiota (Oligo-MM^12^) and Altered Schaedler Flora (ASF)] to better understand the microbial and metabolic dynamics that occur during weaning. Using functional predictions from whole genomes, we measured diversity of metabolic potential, focusing on functions overrepresented in the pre-weaning human microbiome and the nutrients found in human milk. We then interrogated these predictions by comparing the intestinal metabolomic profiles of adult and pre-weaning PedsCom, Oligo-MM^12^, and germ-free mice.

## Results

### Phylogenetic and taxonomic prediction of function from gnotobiotic communities

**Figure 1.**
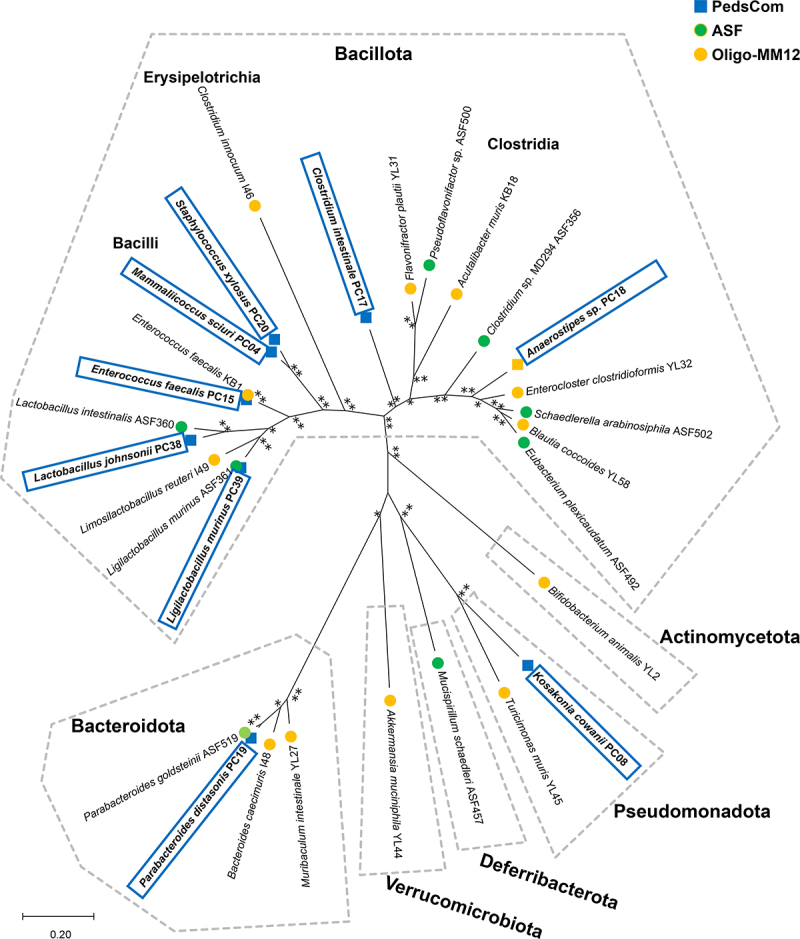
PedsCom consortium models phylogenetic composition of pre-weaning microbiomes.

Phylogenetic and taxonomic diversity can be used to predict the metabolic functions of the microbiome. We first compared PedsCom to two established gnotobiotic consortia, ASF and Oligo-MM^12^, using a phylogenetic approach based on the highly conserved RNA polymerase subunit beta (RpoB) amino acid sequence^15^ (Figure 1 and Table 1). PedsCom contains representatives from three phyla: *Bacillota*, *Bacteroidota* and *Pseudomonadota*, and ASF includes microbes from *Bacillota*, *Bacteroidota* and *Deferribacterota*, while Oligo-MM^12^ is more diverse at the phylum level with the inclusion of the same three phyla in PedsCom as well as the *Actinomycetota* and *Verrucomicrobiota*.

DNA polymerase subunit beta (RpoB) amino acid sequence maximum-likelihood tree of PedsCom, Altered Schaedler Flora (ASF) and Oligo-MM^12^ isolates. The tree with the highest log likelihood is shown. Percentage of bootstrap trees in which associated taxa were clustered represented by **>75%, *>60%.

**Table 1.** Bacterial isolates of PedsCom, altered Schaedler Flora and Oligo-MM^12^ consortia.

Consortium	Genome	Phylum	Class	Order	Family	Genome size (MB)	GC content	KO count	Accession number
PedsCom	*Mammaliicoccus sciuri* PC04	Bacillota	Bacilli	Bacillales	Staphylococcaceae	2.84	32.6	1644	SAMN33929495
	*Staphylococcus xylosus* PC20	Bacillota	Bacilli	Bacillales	Staphylococcaceae	2.83	32.8	1563	SAMN33929501
	*Enterococcus faecalis* PC15	Bacillota	Bacilli	Lactobacillales	Enterococcaceae	2.88	37.5	1466	SAMN33929497
	*Lactobacillus johnsonii* PC38	Bacillota	Bacilli	Lactobacillales	Lactobacillaceae	1.92	34.6	984	GCA_029662885.1
	*Ligilactobacillus murinus* PC39	Bacillota	Bacilli	Lactobacillales	Lactobacillaceae	2.48	39.7	1197	GCA_035904565.1
	*Clostridium intestinale* PC17	Bacillota	Clostridia	Eubacteriales	Clostridiaceae	4.69	30.4	2108	GCA_035905475.1
	*Anaerostipes* sp. PC18	Bacillota	Clostridia	Eubacteriales	Lachnospiraceae	3.39	43.5	1605	SAMN33929499
	*Parabacteroides distasonis* PC19	Bacteroidota	Bacteroidia	Bacteroidales	Tannerellaceae	5.33	45.1	1535	SAMN33929500
	*Kosakonia cowanii* PC08	Pseudomonadota	Gammaproteobacteria	Enterobacterales	Enterobacteriaceae	4.99	55.8	2886	GCA_035928145.1
Altered Schaedler Flora	*Lactobacillus intestinalis* ASF360	Bacillota	Bacilli	Lactobacillales	Lactobacillaceae	1.87	35.9	861	GCA_000364185.2
	*Ligilactobacillus murinus* ASF361	Bacillota	Bacilli	Lactobacillales	Lactobacillaceae	2.11	40.0	1125	GCA_000364205.2
	*Clostridium* sp. ASF356	Bacillota	Clostridia	Eubacteriales	Clostridiaceae	2.81	30.9	1218	GCA_000364165.2
	*Eubacterium plexicaudatum* ASF492	Bacillota	Clostridia	Eubacteriales	Eubacteriaceae	6.10	42.9	1733	GCA_000364225.2
	*Schaedlerella arabinosiphila* ASF502	Bacillota	Clostridia	Eubacteriales	Lachnospiraceae	6.24	47.9	1968	GCA_000364245.2
	*Pseudoflavonifactor* sp. ASF500	Bacillota	Clostridia	Oscillospirales	Oscillospiraceae	3.70	58.8	1274	GCA_000492175.2
	*Parabacteroides goldsteinii* ASF519	Bacteroidota	Bacteroidia	Bacteroidales	Tannerellaceae	6.86	43.4	1594	GCA_000364265.2
	*Mucispirillum schaedleri* ASF457	Deferribacterota	Deferribacteres	Deferribacterales	Mucispirillaceae	2.34	31.2	1274	GCA_000487995.2
Oligo-MM12	*Bifidobacterium animalis* YL2	Actinomycetota	Actinomycetes	Bifidobacteriales	Bifidobacteriaceae	2.02	60.2	919	GCA_002221565.2
	*Enterococcus faecalis* KB1	Bacillota	Bacilli	Lactobacillales	Enterococcaceae	3.03	37.2	1488	GCA_002221625.2
	*Limosilactobacillus reuteri* I49	Bacillota	Bacilli	Lactobacillales	Lactobacillaceae	2.01	38.8	1060	GCA_002221655.1
	*Blautia coccoides* YL58	Bacillota	Clostridia	Eubacteriales	Lachnospiraceae	5.13	45.7	1954	GCA_002221555.2
	*Enterocloster clostridioformis* YL32	Bacillota	Clostridia	Eubacteriales	Lachnospiraceae	6.97	48.1	2252	GCA_002221545.1
	*Acutalibacter muris* KB18	Bacillota	Clostridia	Eubacteriales	Oscillospiraceae	3.80	54.6	1296	GCA_002201475.1
	*Flavonifractor plautii* YL31	Bacillota	Clostridia	Eubacteriales	Oscillospiraceae	3.81	60.9	1558	GCA_002221645.1
	*Clostridium innocuum* I46	Bacillota	Erysipelotrichia	Erysipelotrichales	Erysipelotrichaceae	4.47	43.1	1621	GCA_002221705.2
	*Bacteroides caecimuris* I48	Bacteroidota	Bacteroidia	Bacteroidales	Bacteroidaceae	4.75	42.6	1326	GCA_002221665.1
	*Muribaculum intestinale* YL27	Bacteroidota	Bacteroidia	Bacteroidales	Muribaculaceae	3.31	50.1	1099	GCA_002201515.1
	*Turicimonas muris* YL45	Pseudomonadota	Betaproteobacteria	Burkholderiales	Sutterellaceae	2.89	44.1	1298	GCA_002221595.1
	*Akkermansia muciniphila* YL44	Verrucomicrobiota	Verrucomicrobiae	Verrucomicrobiales	Akkermansiaceae	2.74	55.7	1082	GCA_002201495.1

Listing of consortium isolates investigated in this study. Taxonomic classification to the family level, genome size, GC content and number of annotated KEGG Orthologs (KOs) are included for comparison.

The presence of the *Pseudomonadota* (represented by *Kosakonia cowanii*, formerly *Enterobacter cowanii*) in PedsCom is reflective of mammalian early-life microbiomes, which contain high levels of the *Pseudomonadota* family, *Enterobacteriaceae*. Microbes from this family are among the first colonizers of the human infant gut,^16–18^ and they are major constituents of pre-weaning microbiomes of mice and humans.^5,19^ It has been proposed that facultative anaerobes of the *Enterobacteriaceae* family contribute to the reducing environment needed for colonization of anaerobes with expanded metabolic capabilities.^20,21^ The addition of *K. cowanii* likely increases the proteolytic and lipolytic metabolic capabilities of the PedsCom consortium as these functions are strongly associated with *Pseudomonadota* gut microbes.^22,23^

The *Bacillota* representatives from the three gnotobiotic communities are distributed into three clades representing the classes *Clostridia, Bacilli* and *Erysipelotrichia*. The *Clostridia* clade primarily includes species of the families *Lachnospiraceae* and *Oscillospiraceae* (formerly known as *Ruminococcaceae*). These families comprise a substantial fraction of the adult gut microbiome and support gut homeostasis.^24^ PedsCom contains a single member of *Lachnospiraceae* (*Anaerostipes* sp. PC18), which is in a separate lineage from the representatives in ASF (*Schaedlerella arabinosiphila* ASF502, *Eubacterium plexicaudatum* ASF492) and Oligo-MM^12^ (*Enterocloster clostridioformis* YL32, *Blautia coccoides* YL58). PedsCom does not contain any members of *Oscillospiraceae* (*Flavonifractor*, *Pseudoflavonifractor, Acutalibacter spp*.). Instead, PedsCom contains *Clostridium intestinale*, a *Clostridiaceae*, which has been found in higher abundances in human infants relative to adults.^25^ *Lachnospiraceae* and *Oscillospiraceae* are well-characterized fermenters of host-indigestible fibers into the short-chain fatty acids, propionate and butyrate,^26^ and are enriched in the intestinal mucosa of the adult murine colon.^27^ In contrast, *Clostridiaceae* have a significantly higher potential for proteolytic and lipolytic metabolism than *Lachnospiraceae* and *Oscillospiraceae* species.^22^ Taken together, the taxonomic comparison of the *Clostridia* suggests that critical functions in the adult gut are not recapitulated in PedsCom mice. Indeed, PedsCom mice have much lower levels of short-chain fatty acids in their intestines compared to Oligo-MM^12^ mice.^4^ Instead, the clostridial members of PedsCom are more metabolically suited to utilize protein and lipid substrates compared to their adult-derived counterparts.

*Bacilli*, such as lactobacilli, staphylococci, and enterococci, are a highly prevalent class of bacteria in the pre-weaning microbiomes of mice and humans.^5,19,28^ PedsCom contains two species of *Lactobacillaceae, Lactobacillus johnsonii* and *Ligilactobacillus murinus*. The family *Lactobacillaceae* is quite abundant in the early-life microbiome of mice,^29^ and our recent study^4^ found that *L. johnsonii* is more abundant in the small intestine while *L. murinus* is predominant in the cecum and colon, suggesting niche specialization. *Enterococcus* spp. are also more abundant in gut microbiomes of pre-weaning mice,^30,31^ and the PedsCom and Oligo-MM^12^ *Enterococcus faecalis* isolates are closely related, with an average nucleotide identity (ANI) of 99.1%. PedsCom uniquely contains *Staphylococcaceae* (*Mammaliicoccus sciuri, Staphylococcus xylosus*), which are also more abundant in the pre-weaning microbiomes of mice and humans.^19,31^ Overall, the high proportion and relative abundance of *Bacilli* in PedsCom (5 of 9 taxa, average relative abundance of 71% in small intestine, 26% in large intestine at 2 weeks of age) likely provides many bacterial functions prevalent in the pre-weaning period. These functions include a preference for metabolizing simple sugars over complex carbohydrates and the generation of lactate, which is considerably higher in the gut of pre-weaning infants.^32^

The *Bacteroidota* phylum is prominent in the distal intestines of adult and early-life microbiomes. *Bacteroidota* are well known for their ability to degrade fibers in the host.^33^ PedsCom contains *Parabacteroides distasonis*, which is distantly related to the ASF representative *Parabacteroides goldsteinii*, with an ANI of 74.2% that is at the threshold of separate genera.^34^ The genome of *P. distasonis* is also 30% smaller than *P. goldsteinii’s* (~1.6 MB difference), indicating that *P. goldsteinii* likely encodes many functions absent in *P. distasonis*. Oligo-MM^12^ contains two *Bacteroidota*, *Bacteroides caecimuris* (*Bacteroidaceae*) and *Muribaculum intestinale* of the family *Muribaculaceae*, also known as S24–7. The Oligo-MM^12^ *Bacteroidota* are dominant members of the adult mouse intestinal microbiome.^35^ In contrast, the relative abundance of *P. distasonis* in a complex mature microbial community decreases with age.^4^ Limiting PedsCom to a single pre-weaning associated *Bacteroidota* member likely decreases the fiber-degrading functions found in adult microbial communities.

Altogether the taxonomic configuration of PedsCom predicts several key metabolic differences. First, the unique representation of *Enterobacteriaceae* could favor more proteolytic and lipolytic metabolism. Likewise, the reduction of fiber-degrading *Lachnospiraceae* and *Oscillospiraceae* species, in favor of *Clostridiaceae*, may also favor more proteolytic and lipolytic metabolism. The increased proportion of *Bacilli* species, instead of a *Clostridia- and Bacteroidota*-dominated community, would likely shift carbohydrate metabolism from complex carbohydrates to oligosaccharides. Last, the paucity of *Bacteroidota* in PedsCom and the considerably smaller genome size of *P. distasonis*, the lone PedsCom member of this phylum, suggests that PedsCom may be less able to metabolize complex carbohydrates of a typical adult diet.

### PedsCom consortium is predicted to encode unique functions compared to adult-derived consortia

**Figure 2.**
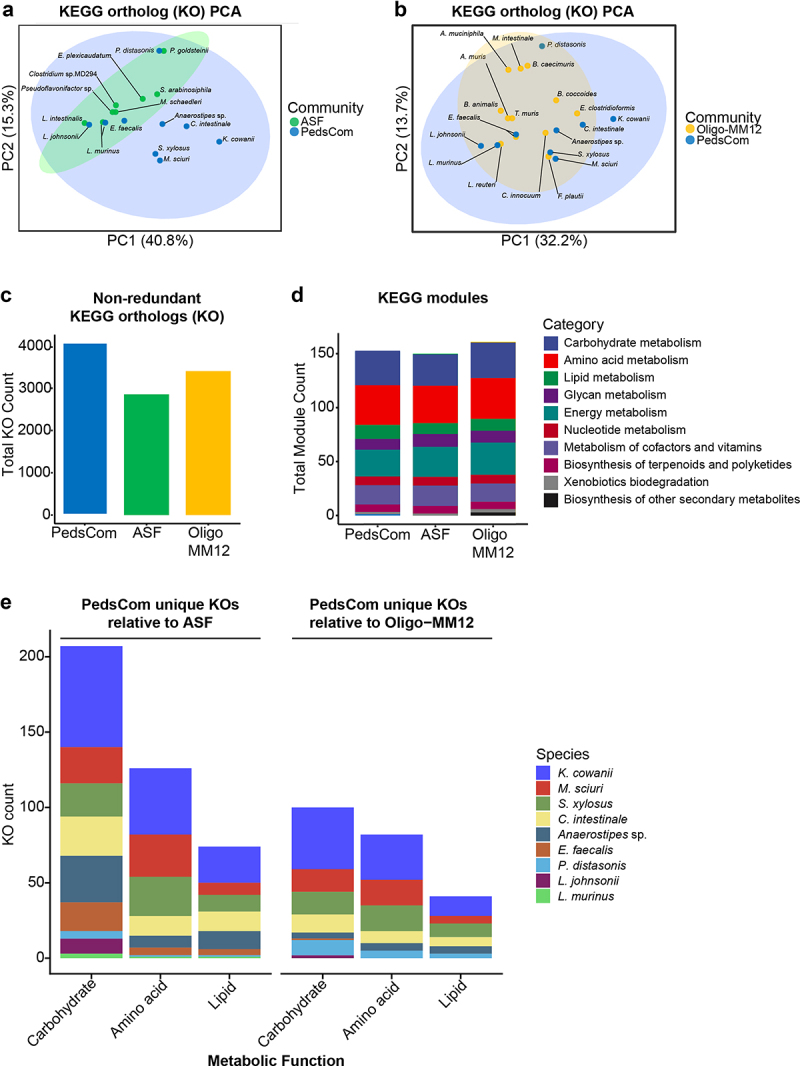
PedsCom consortium isolates are functionally diverse.

To investigate these phylogenetic observations and predictions, we compared the predicted metabolic capabilities of PedsCom to the other two adult-associated consortia using the Kyoto Encyclopedia of Genes and Genomes (KEGG) Orthologs (KOs). Briefly, open reading frames in each genome were assigned to a KO using the KEGG automatic annotation server (KAAS) and combined to represent the total predicted metabolic function of each consortium.^36^ Principal component analysis (PCA) of KOs assigned to KEGG pathways revealed that PedsCom isolates are predicted to be much less functionally related to each other than ASF isolates (Figure 2(a)), denoted by the smaller 95% confidence interval that encompasses ASF isolates relative to PedsCom. These differences in PedsCom are primarily found in the staphylococci (*M. sciuri* and *S. xylosus)*, *Clostridia* (*Anaerostipes* and *C. intestinale)*, and *K. cowanii*. The functional diversity of Oligo-MM^12^ overlapped more with PedsCom, with only *K. cowanii* falling outside the 95% confidence interval of Oligo-MM^12^ (Figure 2(b)).

(a). Principal component analysis (PCA) plots of KEGG Ortholog (KO) pathways of PedsCom and ASF isolates and (b). PedsCom and Oligo-MM^12^ isolates. KO counts in each isolate were aggregated into associated KEGG pathways prior to analysis. Euclidean distances were used to generate PCAs of the functional relatedness of isolates. (c). Non-redundant KOs present in PedsCom, ASF and Oligo-MM^12^ isolates. Total KO count represents the presence of KOs in at least one member of the consortia. Duplicate KOs are only counted once. (d) Number of non-redundant KEGG modules present in PedsCom, ASF, Oligo-MM^12^ consortia, aggregated by functional category. Total module Count represents the presence of KOs in at least one member of the consortia. Duplicate modules are only counted once. (e) Breakdown of unique KOs in PedsCom isolates relative to ASF and Oligo-MM^12^. KEGG orthologs organized by macronutrient metabolism categories. KO count includes KOs shared by PedsCom isolates.

A comparison of absolute KO counts within each consortium revealed that PedsCom (4,062) contains a considerably higher number of KOs than ASF (2,853) or Oligo-MM^12^ (3,403) (Figure 2(c)). We constructed KEGG modules from KOs in each isolate to compare functional capabilities of the three consortia. KEGG modules are functional units of KOs that perform a specific reaction involved in metabolism. Despite the increased numbers of KOs in PedsCom, the numbers of KEGG modules in each consortium were comparable(PedsCom = 152, ASF = 150, Oligo-MM^12^ = 161) (Figure 2(d)). The absolute counts of modules within functional categories were also similar between the three consortia. This suggested some level of functional redundancy in the PedsCom KOs and prompted a closer analysis of the specific modules that set PedsCom apart. Though module counts were similar, the distribution of KOs within module functional subcategories were significantly different between PedsCom and the adult-derived consortia (Χ^2^ test, PedsCom x ASF *p* = 8.439×10^−05^, PedsCom x Oligo-MM12 *p* = 2.148×10^−08^, ASF x Oligo-MM^12^ *p* = 0.527) (Figure S1). Further, PedsCom also devotes more KOs per genome to modules than ASF and Oligo-MM^12^ (median, PedsCom = 456, ASF = 366, Oligo-MM12 = 400) (Table S1). This pattern is particularly evident within carbohydrate metabolism, where PedsCom contains 133 KOs per genome annotated with carbohydrate metabolism, compared to 98 in ASF and 106 in Oligo-MM^12^. This finding initially seemed counterintuitive, as we would have expected adult-derived consortia to contain more metabolic functionality in this category than a pre-weaning consortium, due to the increased diversity of carbohydrate sources post-weaning. However, KEGG modules mostly describe reactions involving carbohydrate monomers and are largely unable to characterize reactions associated with complex carbohydrate degradation such as fiber. Additionally, Euclidean distances of dissimilarity of the three consortia revealed that PedsCom isolates were significantly more dissimilar in their module content than ASF or Oligo-MM^12^ (Figure S2(a)). The higher dissimilarity and higher number of KOs assigned to similar module functions in PedsCom relative to ASF and Oligo-MM^12^ likely account for the higher KO count in PedsCom.

Further investigation of the unique KOs in PedsCom found they included functions uncharacterized by KEGG modules such as transport, genetic information processing, signaling and other cellular processes. When the unique KOs in PedsCom are grouped into major macronutrient metabolic categories (carbohydrates, amino acids, and lipids), carbohydrate metabolism is the most distinct in PedsCom compared to ASF (207 KOs) and Oligo-MM^12^ (100 KOs), but unexpectedly, amino acid metabolism KO profile of PedsCom was almost as distinct (ASF 126 KOs, Oligo-MM^12^ 82 KOs) (Figure 2(e)). The relatively large number of unique amino acid metabolism KOs in PedsCom suggests that PedsCom microbes likely utilize proteins and amino acids differently. Of the PedsCom microbes, *K. cowanii* is the principal contributor of the unique carbohydrate, amino acid and lipid metabolic capabilities, with smaller contributions from the staphylococcal and clostridial species (Figure S2).

To determine if unique KOs present in PedsCom are specifically associated with the functions of the early-life human-associated microbiota, we compared the predicted functions of KOs shared by at least three PedsCom isolates with functions enriched in human infant microbiomes, by comparing these KOs to published human data.^18^ Using this approach, we identified several metabolic functions that are both common and unique to the PedsCom consortium compared to ASF and Oligo-MM^12^ (Table 2). These KOs are primarily associated with metabolism of carbohydrates and lipids, including glycolysis, gluconeogenesis, the TCA cycle, and metabolism of α-linoleic acid, lipoic acid, galactose, glutathione, ubiquinone/menaquinone, fructose and mannose. These findings suggest that many KOs in PedsCom isolates perform metabolic functions that are found in the pre-weaning human microbiome but are absent in the two adult-derived consortia.

**Table 2.** PedsCom unique KOs with functions overrepresented in human infant fecal metagenomes.

KO	KEGG pathway	Description	*Anaerostipes* sp.	*C. intestinale*	*L. johnsonii*	*L. murinus*	*E. faecalis*	*M. sciuri*	*S. xylosus*	*K. cowanii*	*P. distasonis*	Gene count	PedsCom prevlance	Absent in ASF	Absent in OMM12
K02793	Fructose and mannose metabolism	manXa; mannose PTS system EIIA component [EC:2.7.1.191]	4	1	1	2	1	1	0	0	0	10	6/9	X	
K02538	Fructose and mannose metabolism	manR; activator of the mannose operon, transcriptional antiterminator	1	2	1	0	1	2	1	0	0	8	6/9	X	
K02822	Ascorbate and aldarate metabolism	ulaB, sgaB; ascorbate PTS system EIIB component [EC:2.7.1.194]	3	1	0	0	1	1	1	0	0	7	5/9	X	
K02552	Ubiquinone and other terpenoid-quinone biosynthesis	menF; menaquinone-specific isochorismate synthase [EC:5.4.4.2]	0	0	0	0	1	1	1	1	1	5	5/9	X	
K16370	Glycolysis/Gluconeogenesis	pfkB; 6-phosphofructokinase 2 [EC:2.7.1.11]	1	0	1	0	1	0	0	1	0	4	4/9	X	
K00432	Arachidonic acid metabolism	gpx, btuE, bsaA; glutathione peroxidase [EC:1.11.1.9]	0	2	0	0	0	1	1	2	0	6	4/9	X	X
K02768	Fructose and mannose metabolism	fruB; fructose PTS system EIIA component [EC:2.7.1.202]	3	1	0	0	1	1	0	0	0	6	4/9	X	
K02769	Fructose and mannose metabolism	fruAb; fructose PTS system EIIB component [EC:2.7.1.202]	2	1	0	0	1	1	0	0	0	5	4/9	X	
K00383	Glutathione metabolism	GSR, gor; glutathione reductase (NADPH) [EC:1.8.1.7]	0	0	1	0	1	1	0	1	0	4	4/9	X	
K02549	Ubiquinone and other terpenoid-quinone biosynthesis	menC; o-succinylbenzoate synthase [EC:4.2.1.113]	0	0	0	0	1	1	1	1	0	4	4/9	X	
K08680	Ubiquinone and other terpenoid-quinone biosynthesis	menH; 2-succinyl-6-hydroxy-2,4-cyclohexadiene-1-carboxylate synthase [EC:4.2.99.20]	0	0	0	0	1	1	1	1	0	4	4/9	X	
K02744	Galactose metabolism	agaF; N-acetylgalactosamine PTS system EIIA component [EC:2.7.1.-]	2	0	1	0	1	0	0	0	0	4	3/9	X	
K00164	Citrate cycle (TCA cycle)	OGDH, sucA; 2-oxoglutarate dehydrogenase E1 component [EC:1.2.4.2]	0	0	0	0	0	1	1	1	0	3	3/9	X	
K02753	Glycolysis/Gluconeogenesis	ascF; beta-glucoside (arbutin/salicin/cellobiose) PTS system EIICB component [EC:2.7.1.-]	0	0	0	0	0	1	1	1	0	3	3/9	X	
K02779	Glycolysis/Gluconeogenesis	ptsG; glucose PTS system EIICB or EIICBA component [EC:2.7.1.199]	0	0	1	0	1	0	0	1	0	3	3/9	X	
K07406	Galactose metabolism	melA; alpha-galactosidase [EC:3.2.1.22]	0	1	0	0	0	0	1	1	0	3	3/9	X	
K13954	Glycolysis/Gluconeogenesis	yiaY; alcohol dehydrogenase [EC:1.1.1.1]	1	1	0	1	0	0	0	0	0	3	3/9	X	
K16869	Lipoic acid metabolism	lipL; octanoyl-[GcvH]:protein N-octanoyltransferase [EC:2.3.1.204]	0	0	0	0	1	1	1	0	0	3	3/9	X	
K00632	alpha-Linolenic acid metabolism	fadA, fadI; acetyl-CoA acyltransferase [EC:2.3.1.16]	0	0	0	0	0	1	1	2	0	4	3/9	X	
K07160	Glutathione metabolism	pxpA; 5-oxoprolinase (ATP-hydrolyzing) subunit A [EC:3.5.2.9]	0	0	0	0	0	1	1	1	0	3	3/9	X	
K00116	Citrate cycle (TCA cycle)	mqo; malate dehydrogenase (quinone) [EC:1.1.5.4]	0	0	0	0	0	2	2	2	0	6	3/9	X	X
K00138	Glycolysis/Gluconeogenesis	aldB; aldehyde dehydrogenase [EC:1.2.1.-]	0	0	0	0	0	1	1	1	0	3	3/9	X	X
K00241	Citrate cycle (TCA cycle)	sdhC, frdC; succinate dehydrogenase/fumarate reductase, cytochrome b subunit	0	0	0	0	0	1	1	1	0	3	3/9	X	X
K13979	Glycolysis/Gluconeogenesis	yahK; alcohol dehydrogenase (NADP+) [EC:1.1.1.2]	0	0	0	0	0	1	1	1	0	3	3/9	X	X

Most prevalent (shared by ≥ 3 isolates) KEGG Orthologs (KOs) with functions enriched in human infant fecal microbiomes^18^ are presented. Only KOs unique to the PedsCom relative to Altered Schaedler Flora and/or Oligo-MM^12^ are presented.

### Gut-specific metabolic modules reveal a bias toward metabolism of milk-based carbohydrates, amino acids, and lipids in PedsCom

**Figure 3.**
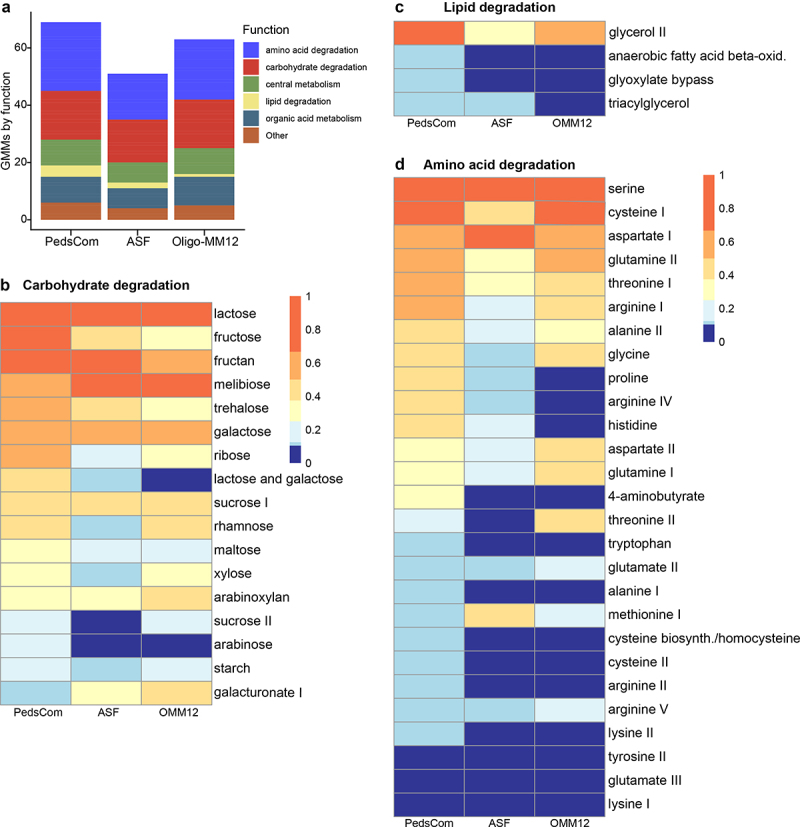
PedsCom consortium encodes a higher capacity for lipid and amino acid degradation.

To investigate the gut-specific metabolic potential of PedsCom compared to ASF and Oligo-MM^12^, we applied a manually curated set of 103 KEGG ortholog modules specific for the gut ecosystem called Gut Metabolic Modules (GMMs).^22^ GMMs accurately assign KOs to processes in the gut and avoid inclusion of superfluous functions that are unlikely to contribute to metabolic functions and fitness in the intestine. To determine the presence or absence of each of the 103 GMMs from each consortium, we set a threshold of possessing at least 75% of the genes assigned to a GMM. Using this criterion, PedsCom has 69 GMMs while ASF has 51 and Oligo-MM^12^ has 63 (Figure 3(a), Table S2). Of the 19 PedsCom GMMs not found in ASF, 18 of them were found in *K. cowanii*, with nine that were exclusive to this microbe. As with the KO analysis above, *Kosakonia cowanii* is the primary contributor of unique GMMs.

(a). Gut Metabolic Modules (GMMs) found in PedsCom, ASF and Oligo-MM^12^ isolates. GMMs are considered present if at least 75% of the KEGG orthologs associated with module were found in an isolate. (b) Heatmap of the proportion of PedsCom, ASF and Oligo-MM^12^ isolates that contain carbohydrate degradation GMMs. (c) Heatmap of the proportion of PedsCom, ASF and Oligo-MM^12^ isolates that contain lipid degradation GMMs. (d) Heatmap of the proportion of PedsCom, ASF and Oligo-MM^12^ isolates that contain amino acid degradation GMMs. Heatmap rows are ordered by proportion of PedsCom microbes which contain the module.

Because the GMM database focuses mostly on mono- and disaccharide degradation capabilities that are common to oligo and polysaccharides alike, carbohydrate degradation modules are expected to appear similar across the three consortia regardless of diet. Beta-galactosidases that degrade lactose (MF0006) were widespread among the consortia, present in all PedsCom isolates and 75% of ASF and Oligo-MM^12^ (Figure 3(b) and Table S2). However, PedsCom had a higher proportion of lactose and galactose module (MF0007) containing isolates, compared to ASF and Oligo-MM^12^. MF0007 is almost exclusively found in *Lactobacillales* and *Collinsella* species and includes a phosphotransferase system transporter for lactose and D-tagatose-6-phosphate pathway of galactose degradation.^22^ *Lactobacillales* and *Collinsella* are clades abundant in the pre-weaning microbiome, suggesting that the presence of MF0007 could provide a nutritional advantage in the pre-weaning gut. The PedsCom consortium also has fewer members that can degrade fibers found in solid food diets including galacturonate and arabinoxylan, which are constituents of pectin and hemicellulose fibers, respectively (Figure 3(b) and Table S2).

To further survey carbohydrate metabolism, we investigated the predicted ability of PedsCom isolates to degrade host-indigestible milk oligosaccharides (MOs). Humans produce a complex mixture of fucosylated, sialylated, and nonfucosylated neutral MOs, with the most abundant being 2-fucosyllactose.^37^ Mouse milk does not contain fucosylated MOs, instead the most abundant milk oligosaccharides are sialyllactoses (SLs), which consist of a lactose molecule bound to sialic acid by either an α2–6 (6’-SL) or α2–3 linkage (3’-SL).^38^ Human milk oligosaccharide (HMO) consumption is primarily linked with *Bifidobacterium* and *Bacteroides* species in the infant gut.^39^ The PedsCom isolate *P. distasonis* contains orthologous carbohydrate metabolism gene clusters that are up-regulated in *Bacteroides* species during growth on HMOs, including 6’-SL and 3’-SL.^40^ These genes include a sialidase (*nanH*) as well as a complete sialic acid catabolic (*nan*) operon (Table S3). A typical *nan* gene cluster contains *N-*acetylneuraminate lyase gene (*nanA), N-*acetylmannosamine 2-epimerase gene (*nanE*), and *N*-acetylmannosamine kinase gene (*nanK*), though *Bacteroidota* species are able to catabolize sialic acid without *nanK* .^41,42^ These findings suggest that *P. distasonis* can cleave sialic acid from sialyllactose and also use sialic acid as a carbon and nitrogen source. While none of the other PedsCom members possess a *nanH* sialidase gene, two other members of PedsCom, *Anaerostipes* sp. and *S. xylosus*, contain a putative *nan* cluster (Table S3) suggesting that they can metabolize sialic acid liberated by *P. distasonis* sialidase activity. *Blautia coccoides* was the only non-*Bacteroidota* Oligo-MM^12^ isolate with a complete *nan* cluster, while no non-*Bacteroidota* ASF isolates contained a complete cluster. Interestingly, 8 of 9 PedsCom isolates contained a putative *nanE* gene compared to 3 of 8 ASF isolates and 3 of 12 Oligo-MM^12^, suggesting a preference toward amino sugar metabolism in the consortium. In conclusion, further genomic analysis suggests that the PedsCom consortium can putatively consume the predominant milk oligosaccharides found in mouse milk.

The lipid fraction of milk is organized into milk fat globules consisting of a triglyceride core surrounded by phospholipid and sphingolipid layers.^43^ Lipid degradation modules are also more prevalent within PedsCom with 7 of 9 PedsCom isolates capable of glycerol degradation (Figure 3(c)). Metabolizing glycerol to glyceraldehyde 3-phosphate allows shuttling of free glycerol liberated from milk triglycerides by host lipase activity into the central metabolic pathways of these microbes.^44^ In PedsCom, *K. cowanii* uniquely encodes the putative abilities of anaerobic beta-oxidation of medium and long-chain fatty acids (MF0059) to generate acetyl-CoA by utilizing the glyoxylate bypass pathway (MF0063) to convert acetyl-CoA to TCA cycle intermediates, such as succinate (Table S2). Both MF0059 and MF0063 functions are associated with gut microbiome *Pseudomondata*,^22^ though beta-oxidation among gut microbes is otherwise rare due to oxygen typically being used as the terminal electron acceptor during the process. However, our PedsCom KO analysis indicated that *S. xylosus* and *M. sciuri* could likely also perform fatty acid beta-oxidation. With the nitrate reductase *narGHJI* operon found in both of these species, PedsCom staphylococcal isolates could potentially perform anaerobic beta-oxidation using nitrate as the terminal electron acceptor, as seen in other staphylococci.^45^ Additionally, PedsCom microbes seem predisposed toward phospholipid metabolism. Seven of nine PedsCom isolates encode a putative phospholipase, with *K. cowanii* encoding the highest number of these genes. The high prevalence of phospholipases in PedsCom isolates suggests an important role for phospholipids in community-wide metabolism.

Amino acid degradation (AAD) GMMs are the dominant unique functions found in PedsCom (Figure 3(d)). Of the 27 AAD GMMs present in the three consortia, PedsCom has 24. Six AAD GMMs were exclusively found in PedsCom. The most differentially abundant AAD modules in PedsCom relative to ASF and Oligo-MM^12^ were: γ-aminobutyrate (GABA) (found in 3 of 9 PedsCom isolates, in none of the ASF or Oligo-MM^12^ isolates), proline (PedsCom = 4/9, ASF = 1/8, Oligo-MM^12^ = 1/12), and histidine (PedsCom = 4/9, ASF = 2/8, Oligo-MM^12^ = 1/12). GABA metabolism and transporters for proline and histidine are significantly associated with the pre-weaning microbiome of human infants.^18^ Arginine metabolism was also differentially abundant between the consortia (Figure 3(d)). PedsCom contains 6 of 9 isolates with arginine GMMs, compared to 2 of 8 ASF isolates and 7 of 12 Oligo-MM^12^. Though the proportion of arginine GMMs containing isolates was similar between PedsCom and Oligo-MM^12^, arginine degradation is more diverse within PedsCom. Five PedsCom isolates contained multiple pathways of arginine degradation, while only one Oligo-MM^12^ isolate contained multiple pathways. The prevalence of arginine degradation strategies among PedsCom isolates suggests that arginine is important among early-life associated microbes, a finding that is supported by a significant association between the abundance of arginine metabolism and transport genes in human infant microbiomes.^18^ Notably, the PedsCom members *K. cowanii* (*n* = 18 GMMs) and *P. distasonis* (*n* = 15 GMMs) had the most AAD GMMs of any isolate in the three consortia (median per isolate = 6 GMMs) (Table S2).

Of the 13 PedsCom GMMs not found in Oligo-MM^12^, nine are either amino acid or lipid degradation modules. We observed a similar pattern in PedsCom GMMs absent in ASF, in which 10 of the 19 absent functions are involved in amino acid or lipid degradation. This pattern indicates that the PedsCom consortium is poised for increased protein and lipid consumption.

### PedsCom depletes milk-associated amino acids in vivo

**Figure 4.**
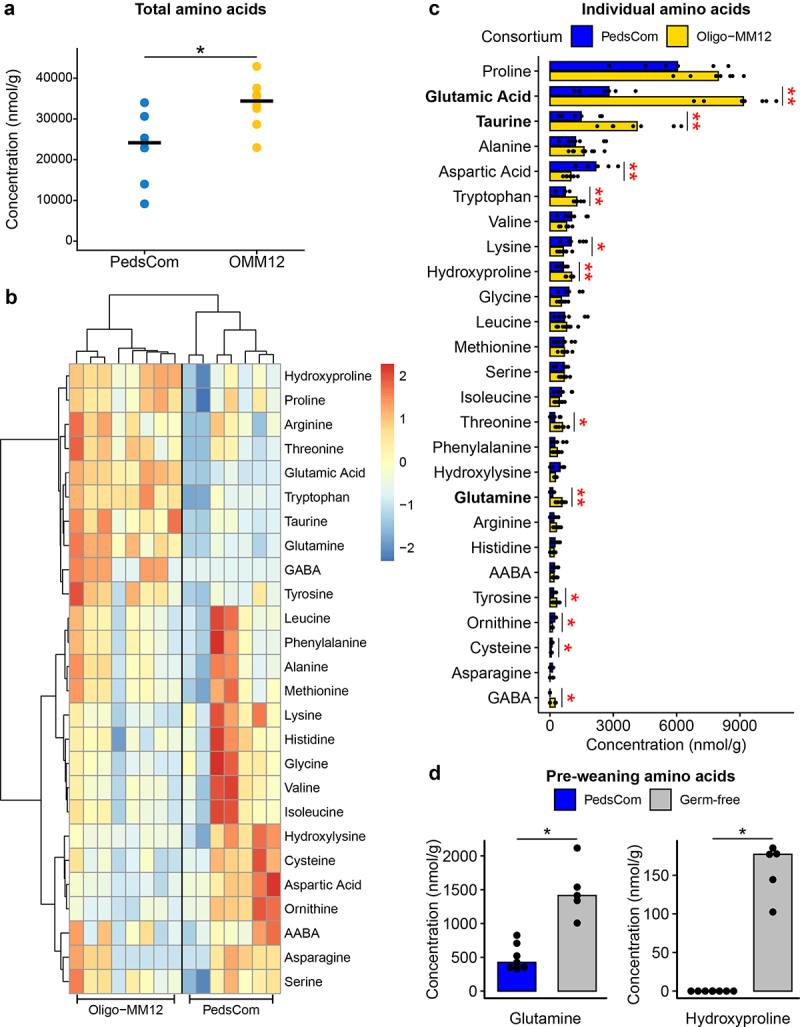
PedsCom colonized mice have reduced amino acid levels in the gut.

The higher abundance of amino acid degradation modules in PedsCom relative to Oligo-MM^12^ led us to hypothesize that there would be lower levels of free amino acids in the gut of PedsCom mice. To test this hypothesis, we performed metabolomic analysis on cecal contents of adult PedsCom- and Oligo-MM^12^-colonized mice fed standard mouse chow. In support of the hypothesis, we found significantly lower levels of free amino acids in PedsCom mice compared to Oligo-MM^12^ (24,146 vs 33,278 nmol/g, *p* < .05) (Figure 4(a)). Hierarchical clustering of amino acid levels in the cecum revealed two clusters of high and low abundance amino acids in the gut of PedsCom mice (Figure 4(b)). The top three most abundant free amino acids in milk (glutamic acid, taurine, glutamine)^11^ were significantly depleted in PedsCom cecal contents, relative to Oligo-MM^12^ colonized mice (Figure 4(c)). From these analyses, we conclude that PedsCom depletes milk-associated amino acids in the gut, which supports the prediction that PedsCom has a greater capacity to metabolize amino acids.

(a). Total free amino acid levels (nmol/g) in cecal contents of PedsCom (*n* = 7) and Oligo-MM^12^ (*n* = 8) colonized mice. (b) Heatmap of free amino acids levels z-score normalized across samples. Hierarchal clustering of columns and rows performed by Ward1 clustering method and Euclidean distance. (c) Individual amino acid levels (nmol/g) in cecal contents of adult PedsCom and Oligo-MM^12^ mice. The three most abundant free amino acids found in milk are bolded. (d) Free glutamine and hydroxyproline levels in fecal contents of pre-weaning PedsCom (*n* = 7) and germ-free mice (*n* = 5). Bar plots represent median values. False discovery rate (FDR) corrected, Mann-Whitney-Wilcoxon tests performed, *p < .05, **p < .01, ***p < .001.

To test how PedsCom amino acid metabolism functions under an exclusively milk-based host diet, the feces of 11–13-day-old PedsCom and germ-free mice were analyzed for free amino acid levels. While there was no significant difference in median total levels of free amino acids in pre-weaning PedsCom and germ-free mice fecal samples (10.153 vs 8.939 nmol/g, *p* = .53), hierarchical clustering of individual amino acid levels clearly separated the two sample types (Figure S3). Two amino acids (glutamine, hydroxyproline) were significantly depleted in PedsCom mice relative to germ-free mice, mirroring the depletion of glutamine and hydroxyproline by PedsCom isolates in adult mice (Figure 4(c,d)).

## Discussion

We compared the phylogenetic and functional metabolic features of an early-life gnotobiotic community with two adult gnotobiotic communities to better understand the dynamic transition between pre-weaning and post-weaning microbiomes. We made two key observations. First, the predicted metabolic functions of PedsCom are well-matched to the nutrient profile of milk from the standpoint of carbohydrates, proteins, and lipids. Second, we found a strong predicted metabolic signal for amino acid degradation pathways in the PedsCom community and corroborated this prediction with *in vivo* metabolomic analysis. This skew in PedsCom metabolism is consistent with the functions present in human infant microbiota, highlighting the impact of the milk-based diet on pre-weaning commensal metabolism.

While PedsCom is predicted to be proteolytic, a sizable proportion (~25%) of the available nitrogen in milk comes from non-protein sources such as free amino acids.^46^ Free amino acids could provide a readily available source of additional nitrogen and energy to pre-weaning gut commensals that are typically unavailable in solid food. Our metabolic prediction is supported by our data showing that PedsCom colonized adult mice were depleted of free amino acids, particularly those most commonly found in milk, relative to Oligo-MM^12^ controls. This prediction is further supported by significant depletion of glutamine and hydroxyproline in pre-weaning PedsCom mouse intestines compared to germ-free controls.

Amino acid metabolism has the potential to shape the preweaning microbiota and host immune development during the critical weaning transition. In addition, differences in amino acid utilization between gnotobiotic consortia like PedsCom and Oligo-MM^12^ could have downstream consequences for host cell function and immunity potentially through the interconversion of dietary amino acids as well as production of biogenic amines, phenolic and indolic compounds.^47–49^ Several amino acids impacted by PedsCom colonization (glutamic acid, glutamine, GABA) have immunomodulatory and cellular signaling functions in the host gut,^50,51^ suggesting that the PedsCom gnotobiotic model can serve as system to study the impact of microbial metabolism on the host during the critical window around weaning.

## Methods

### Mice

Germ-free NOD mice were housed at the Hill Pavilion gnotobiotic mouse facility, University of Pennsylvania, in flexible film isolators [Class Biologically Clean (CBClean)]. Adult mice were transferred to sterile cages prior to colonization with the PedsCom and Oligo-MM^12^ consortia. Mice were fed LabDiet 5021 (Cat# 0006540) and caged on autoclaved beta-chip hardwood bedding (Nepco). Animal studies were approved by the Institutional Animal Care and Use Committee (IACUC) of the University of Pennsylvania.

### Phylogenetic analysis

RNA polymerase subunit beta (RpoB) amino acid sequences of PedsCom, ASF and Oligo-MM^12^ isolates were aligned with MAFFT (ver. 7.243) using the E-INS-I method.^52^ Amino acid substitution models were fitted to the alignment and the L_Gasecuel_2008 with discrete Gamma distribution (LG+G) model with complete deletion was used to generate a maximum likelihood phylogenetic tree of the consortia members in MEGA X.^53^ Average nucleotide identity (ANI) was calculated using the OrthoANIu algorithm.^54^

### Predicted metabolic function analysis

Annotated whole-genome sequences of PedsCom isolates were generated as previously described.^4^ Genome files for ASF and Oligo-MM^12^ isolates were obtained from the Bacterial and Viral Bioinformatics Resource Center (BV-BRC). Open reading frames in each isolate were assigned a KEGG Ortholog designation through the KEGG automatic annotation server (KAAS).^36^ Euclidian distance measurements used in PCA plots of isolate KO counts per KEGG pathway were generated using the MicrobiomeAnalyst R package.^55^ KEGG modules were assigned using the MicrobiomeAnalyst R package. KEGG modules were annotated into functional classes, categories and subcategories manually using module descriptions obtained from the KEGG database. Differential analysis of the distribution of KOs in KEGG modules by functional subcategory was performed by pairwise Χ^2^ tests of each consortium. Dissimilarity indices of KO counts within KEGG modules were determined by median Euclidean distances between isolates within each consortium. Modules found in more 80% of the isolates (>23) were removed to limit uninformative functions prior to differential and dissimilarity analyses. The functions of unique PedsCom KOs not associated with KEGG modules were determined by BRITE hierarchy classification using the Reconstruct tool of the KEGG Mapper webservice. Functional comparisons of unique KOs commonly detected (≥3 isolates) in PedsCom to KEGG pathways enriched in pre-weaning human infant microbiomes were performed on published shotgun metagenomic data.^18^ The dataset consisted of 98 maternal fecal samples collected at delivery and longitudinal full-term infant fecal samples collected a few days after birth (newborn), and at 4 and 12 months of age. Only newborn and 4-month-old data were considered as pre-weaning timepoints. KEGG pathways enriched in infants relative to the adults (maternal samples) were determined by the reporter score algorithm.^56^ Only pathways with reporter scores < −1.6 were considered significantly enriched in pre-weaning microbiomes. Gut Metabolic Modules^22^ were downloaded through github: https://github.com/raeslab/GMMs, and isolate KOs were assigned to a GMM using ANVI’O.^57^ A module was considered present if 75% of the genes required for the module were detected in the isolate. Among the consortia, the prevalence of GMM modules was determined by the proportion of isolates containing each module.

### Intestinal amino acid concentration analysis

PedsCom and Oligo-MM^12^ isolates were cultured and gavaged into 5–6-week-old germ-free C57BL/6J mice as previously described.^4^ Colonized mice were housed in sterile cages and sacrificed after 2 weeks to collect cecal contents for analysis. Examination of free amino acids in 10–13-day-old PedsCom and germ-free mice was performed to determine amino acid metabolism under a milk-based diet. Pups were housed with their dams immediately before sacrifice and collection of fecal contents. Intestinal samples were flash frozen at −80°C, prior to metabolomic analysis. Free amino acid concentrations were measured by the PennCHOP microbiome core using the Waters Acuity UPLC system (Cat# 176015000) with an AccQ-Tag Ultra C18 1.7 μm 2.1 × 100 mm column and a photodiode detector array (Waters, Cat# 186003837). Analysis was performed using the UPLC AAA H-Class Application Kit (Waters, Cat# 176002983). Limit of detection for individual amino acids was 1 nmol/g cecal contents.

### Statistical analysis

Heatmaps were generated with the R package pheatmap. Amino acid concentrations were z-score normalized across rows prior to heatmap generation. Hierarchical clustering of columns and rows in amino acid heatmaps was performed by the Ward1 clustering method using Euclidean distances. Statistical analyses were performed in R using base R and the ggpubr package. Differences in amino acid levels were determined by Mann-Whitney-Wilcoxon tests on median values with p-values <0.05 considered significant. p-values were corrected by false discovery rate to control for multiple comparisons. Kruskal–Wallis Rank test and Dunn’s tests of multiple comparisons were performed on Euclidean distance indices of KEGG module subcategories.

## Supplementary Material

Supplemental Material

## Data Availability

All data associated with this study are available upon request.
