# Supplementary figures and images for: Comparison of gnotobiotic communities reveals milk-adapted metabolic functions and unexpected amino acid metabolism by the pre-weaning microbiome
Source: Gut Microbes. 2024 Aug 12;16(1):2387875. doi: 10.1080/19490976.2024.2387875 (PMC11321411; doi:10.1080/19490976.2024.2387875)

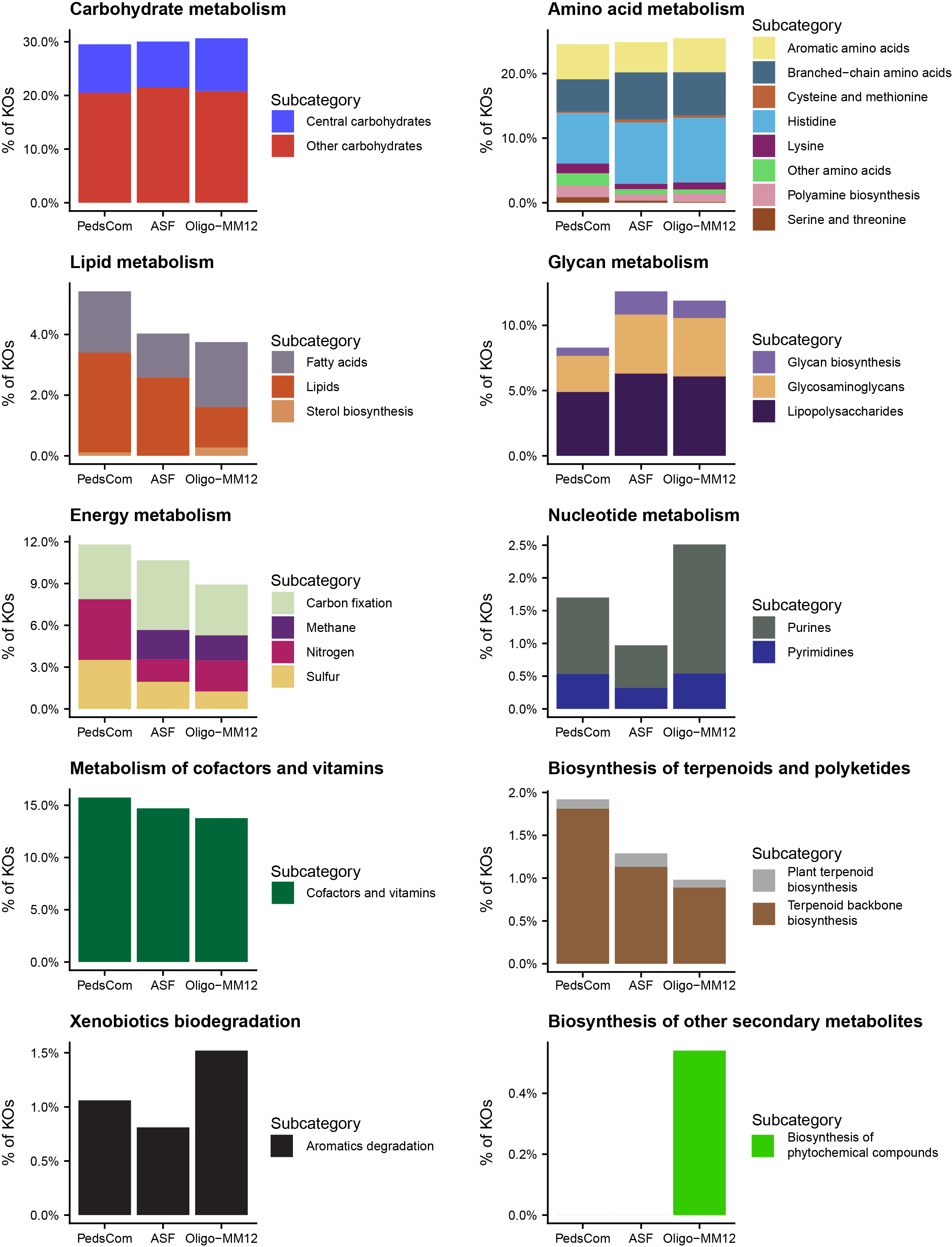

Supplement: Supplemental Material [file KGMI_A_2387875_SM0674.zip › Supplementary Material/FigS1_KGMI_20231190.tif]

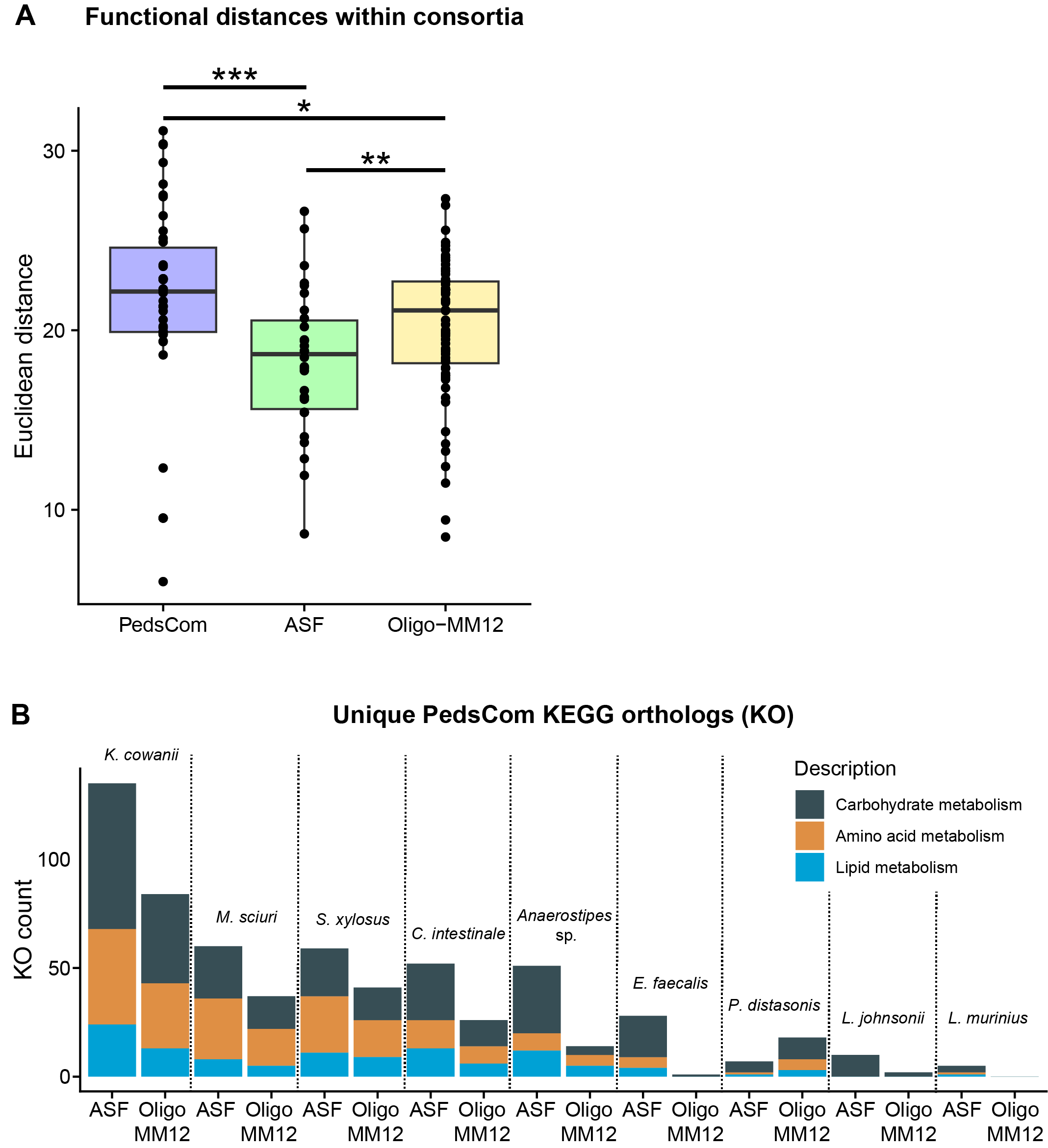

Supplement: Supplemental Material [file KGMI_A_2387875_SM0674.zip › Supplementary Material/FigS2_KGMI_20231190.tif]

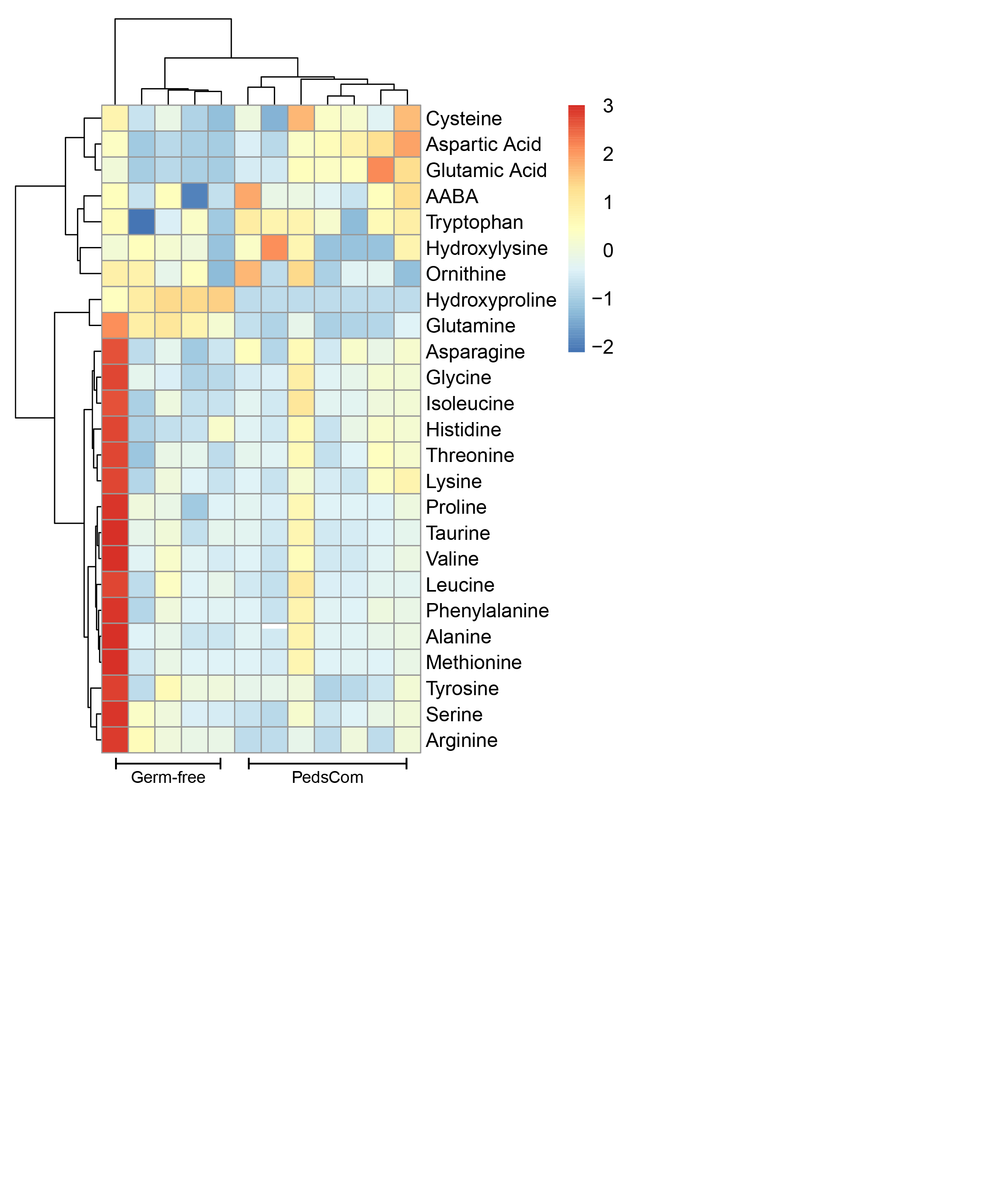

Supplement: Supplemental Material [file KGMI_A_2387875_SM0674.zip › Supplementary Material/FigS3_KGMI_20231190.tif]
